# COVID-19 Mortality Among American Indian and Alaska Native Persons — 14 States, January–June 2020

**DOI:** 10.15585/mmwr.mm6949a3

**Published:** 2020-12-11

**Authors:** Jessica Arrazola, Matthew M. Masiello, Sujata Joshi, Adrian E. Dominguez, Amy Poel, Crisandra M. Wilkie, Jonathan M. Bressler, Joseph McLaughlin, Jennifer Kraszewski, Kenneth K. Komatsu, Xandy Peterson Pompa, Megan Jespersen, Gillian Richardson, Nicholas Lehnertz, Pamela LeMaster, Britney Rust, Alison Keyser Metobo, Brooke Doman, David Casey, Jessica Kumar, Alyssa L. Rowell, Tracy K. Miller, Mike Mannell, Ozair Naqvi, Aaron M. Wendelboe, Richard Leman, Joshua L. Clayton, Bree Barbeau, Samantha K. Rice, Victoria Warren-Mears, Abigail Echo-Hawk, Andria Apostolou, Michael Landen

**Affiliations:** ^1^Council of State and Territorial Epidemiologists, Atlanta, Georgia; ^2^Northwest Portland Area Indian Health Board, Portland, Oregon; ^3^Urban Indian Health Institute, Seattle Washington; ^4^Alaska Department of Health and Social Services; ^5^Albuquerque Area Indian Health Board, Albuquerque, New Mexico; ^6^Arizona Department of Health Services; ^7^Louisiana Department of Health; ^8^Minnesota Department of Health; ^9^Mississippi State Department of Health; ^10^Nebraska Department of Health and Human Services; ^11^New Mexico Department of Health; ^12^New York State Department of Health; ^13^North Dakota Department of Health; ^14^Oklahoma State Department of Health; ^15^Oregon Health Authority; ^16^South Dakota Department of Health; ^17^Utah Department of Health; ^18^Washington State Department of Health; ^19^Indian Health Service, Rockville, Maryland

American Indian/Alaska Native (AI/AN) persons experienced disproportionate mortality during the 2009 influenza A(H1N1) pandemic (*1*,*2*). Concerns of a similar trend during the coronavirus disease 2019 (COVID-19) pandemic led to the formation of a workgroup* to assess the prevalence of COVID-19 deaths in the AI/AN population. As of December 2, 2020, CDC has reported 2,689 COVID-19–associated deaths among non-Hispanic AI/AN persons in the United States.^†^ A recent analysis found that the cumulative incidence of laboratory-confirmed COVID-19 cases among AI/AN persons was 3.5 times that among White persons (*3*). Among 14 participating states, the age-adjusted AI/AN COVID-19 mortality rate (55.8 deaths per 100,000; 95% confidence interval [CI] = 52.5–59.3) was 1.8 (95% CI = 1.7–2.0) times that among White persons (30.3 deaths per 100,000; 95% CI = 29.9–30.7). Although COVID-19 mortality rates increased with age among both AI/AN and White persons, the disparity was largest among those aged 20–49 years. Among persons aged 20–29 years, 30–39 years, and 40–49 years, the COVID-19 mortality rates among AI/AN were 10.5, 11.6, and 8.2 times, respectively, those among White persons. Evidence that AI/AN communities might be at increased risk for COVID-19 illness and death demonstrates the importance of documenting and understanding the reasons for these disparities while developing collaborative approaches with federal, state, municipal, and tribal agencies to minimize the impact of COVID-19 on AI/AN communities. Together, public health partners can plan for medical countermeasures and prevention activities for AI/AN communities.

During July 22–September 3, 2020, data were collected on confirmed COVID-19–associated deaths that occurred during January 1–June 30, 2020, from 14 participating states.^§^ These states represent approximately 46.5% of the AI/AN population in the United States.^¶^ States provided data on confirmed COVID-19 deaths by two race/ethnicity groups (AI/AN and White), sex (men and women), and 10-year age groups. At the request of the participating tribal epidemiology centers and states, White race was chosen as the sole comparator to avoid comparison of AI/AN persons of other races/ethnicities that have experienced similar COVID-19 health disparities. AI/AN race was defined as AI/AN either alone or in any racial/ethnic combination; White race was defined as non-Hispanic White only.** The workgroup, which included epidemiologists and tribal epidemiology subject matter experts, also collected data on underlying health conditions known to increase risk for severe COVID-19–associated illness; however, incomplete data precluded analysis of underlying health conditions. Data for race, ethnicity, and COVID-19 mortality were obtained by the state health departments from multiple sources, including case investigations, death certificates, and laboratory reports. Age-adjusted and age-specific COVID-19 mortality rates were calculated for AI/AN and White populations.^††^ The AI/AN and White populations for each state were obtained from 2019 postcensal population estimates^§§^ and used as the denominator for rate calculations. Death rates by race/ethnicity were age-adjusted to the 2000 U.S. standard population; 95% CIs for rates were calculated using the Byar approximation to the Poisson distribution. COVID-19 death rates among AI/AN were compared with those among White persons using rate ratios. The number of deaths among persons aged <20 years was small (<10), and these data were suppressed to avoid possible harm to AI/AN communities if potentially identifiable data were published. This activity was reviewed by the Council of State and Territorial Epidemiologists and was conducted for public health surveillance purposes consistent with applicable federal law.^¶¶^

Participating states reported 1,134 deaths among AI/AN persons and 18,815 deaths among White persons during January 1–June 30, 2020. Men accounted for 621 (55%) AI/AN deaths and 9,775 (52%) deaths in White persons. Overall, AI/AN persons who died from COVID-19 were younger than were White persons: 35.1% of AI/AN COVID-19–associated deaths were among persons aged <60 years, compared with 6.3% of deaths among White persons (Table) (Figure).

**TABLE:** COVID-19–associated deaths* among American Indian/Alaska Native (AI/AN) ^†^ and non-Hispanic White (White) persons aged ≥20 years,^§^ by demographic characteristics — 14 states,^¶^ January–June 2020

Characteristic	AI/AN deaths		White deaths		AI/AN:White rate ratio (95% CI)
	No. (%)**	Rate^††^ (95% CI)	No. (%)^§§^	Rate^††^ (95% CI)	
Total*	**1,134 (100)**	**55.8 (52.5–59.3)**	**18,815 (100)**	**30.3 (29.9–30.7)**	**1.8 (1.7–2.0)**
Sex*					
Men	621 (55)	66.4 (60.9–72.1)	9,775 (52)	36.1 (35.4–36.8)	1.8 (1.7–2.0)
Women	513 (45)	46.8 (42.8–51.1)	9,035 (48)	25.4 (24.9–26.0)	1.8 (1.7–2.0)
Missing	0	—	5	—	—
**Age group, yrs^¶¶^**					
20–29	27 (2)	6.3 (4.2–9.2)	31 (0.2)	0.6 (0.4–0.9)	10.5 (6.3–17.6)
30–39	72 (6)	19.8 (15.5–25.0)	91 (0.5)	1.7 (1.4–2.1)	11.6 (8.5–15.8)
40–49	99 (9)	34.0 (27.6–41.3)	199 (1)	4.1 (3.6–4.7)	8.2 (6.5–10.5)
50–59	200 (18)	73.9 (64.1–84.9)	870 (5)	15.6 (14.6–16.7)	4.7 (4.1–5.5)
60–69	268 (24)	127.7 (112.8–143.9)	2,337 (12)	41.6 (39.9–43.3)	3.1 (2.7–3.5)
70–79	235 (21)	218.1 (191.1–247.8)	4,514 (24)	122.2 (118.7–125.9)	1.8 (1.6–2.0)
≥80	230 (20)	488.3 (427.3–555.7)	10,767 (57)	520.1 (510.3–530.0)	0.9 (0.8–1.1)
Missing	3	—	6	—	—

**Abbreviations:** CI = confidence interval; COVID-19 = coronavirus disease 2019.

* Rates are age-adjusted to the 2000 U.S. standard population.

^†^ Includes Hispanic and non-Hispanic AI/AN persons.

^§^ Cases in persons aged <20 years suppressed because number was <10.

^¶^ Alaska, Arizona, Louisiana, Minnesota, Mississippi, Nebraska, New Mexico, New York, North Dakota, Oklahoma, Oregon, South Dakota, Utah, and Washington.

** Includes 12 persons with unknown ethnicity.

^††^ Deaths per 100,000 population.

^§§^ Includes five persons with unknown sex.

^¶¶^ Rates are age-specific.

**FIGURE:**
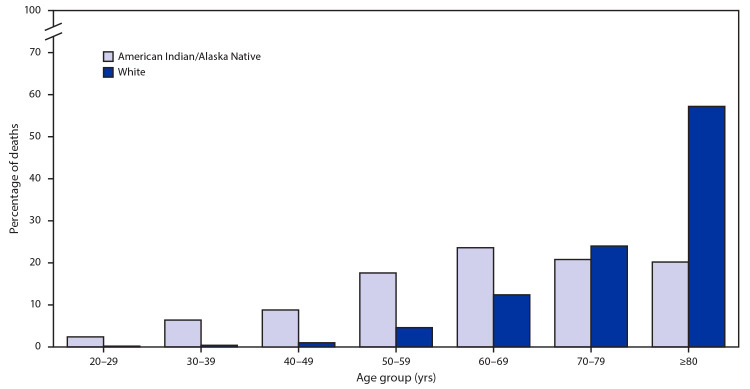
Percentage distribution of COVID-19–associated deaths among American Indian/Alaska Native* and non-Hispanic White persons aged ≥20 years, by age group^†^ — 14 states,^§^ January 1–June 30, 2020 **Abbreviation:** COVID-19 = coronavirus disease 2019. * Includes Hispanic and non-Hispanic ethnicities. ^†^ Percentages by age group are not age-adjusted. ^§^ Alaska, Arizona, Louisiana, Minnesota, Mississippi, Nebraska, New Mexico, New York, North Dakota, Oklahoma, Oregon, South Dakota, Utah, and Washington.

The age-adjusted AI/AN COVID-19 mortality rate (55.8 deaths per 100,000) was 1.8 (95% CI = 1.7–2.0) times that among White persons (30.3 deaths per 100,000) (Table). For both AI/AN and White persons, mortality was higher among men (66.4 and 36.1 per 100,000, respectively) than among women (46.8 and 25.4 per 100,000, respectively). Among both AI/AN and White persons, COVID-19 mortality rates increased with age, with the highest age-specific mortality rate among persons aged ≥80 years (488.3 and 520.1 per 100,000, respectively). Mortality rates among AI/AN persons were higher than those among White persons in all age groups except the oldest age group. The largest differences were among persons in the three age groups encompassing 20–49 years. Among persons aged 20–29 years, 30–39 years, and 40–49 years, the COVID-19 mortality rates among AI/AN persons were 10.5, 11.6, and 8.2 times those among White persons, respectively (Table).

## Discussion

In the 14 states that participated in this study, the overall COVID-19 mortality rate among AI/AN persons was higher than that among White persons. This finding is consistent with those from a similar study assessing pandemic influenza A(H1N1)–related mortality (*1*). Long-standing inequities in public funding; infrastructure; and access to health care, education, stable housing, healthy foods, and insurance coverage have contributed to health disparities (including higher prevalences of smoking, obesity, diabetes, and cardiovascular disease) that put indigenous peoples at higher risk for severe COVID-19–associated illness (*4*). The lack of consistent and complete collection of underlying health conditions prevented the workgroup from assessing the contributions of these conditions to the observed disparity in mortality. This highlights the need for consistent approaches across jurisdictions to collect this information systematically and completely. As with influenza mortality rates, differences in socioeconomic factors might have contributed to elevated COVID-19 mortality (*5*). Financial or transportation-related barriers to health care access might have prevented patients from receiving timely medical care at the time of initial evaluation, resulting in more severe illness that was less amenable to treatment (*2*).

In 2010, the Advisory Committee on Immunization Practices (ACIP) first recommended that vaccination efforts should focus on delivering influenza vaccine to AI/AN population, among others, when supply is limited, based on the disproportionate impact pandemic influenza A(H1N1) had on AI/AN communities during 2009–2010 (*6*). The ACIP COVID-19 Vaccines Work Group has developed an ethical framework to guide COVID-19 vaccine allocation decisions when supply is limited (*7*), which aims to maximize benefits and minimize harms, promote justice, mitigate health inequities, and promote transparency. Compared with White persons, AI/AN persons have experienced higher morbidity and mortality from COVID-19 (*3*).*** Federal, state, tribal, and local partners should consider the AI/AN disparities from COVID-19 and other underlying factors when developing their vaccine allocations strategies.

The findings in this report are subject to at least six limitations. First, mortality estimates for other persons of color were not assessed, preventing comparisons with these groups. Second, deaths caused by COVID-19 were likely underreported because of limited testing availability and reluctance to be tested, particularly in the early months of the pandemic (*8*). Third, limited completeness and accuracy of race/ethnicity data might lead to undercounting of AI/AN COVID-19–related deaths. AI/AN persons are more likely to be racially misclassified as White or other races in vital records and other data systems, resulting in underestimates of morbidity and mortality in AI/AN communities (*9*). Fourth, the inconsistent and incomplete collection of data for underlying health conditions precluded an analysis controlling for underlying health conditions as a factor for COVID-19 mortality. Fifth, the analytic methods used did not account for clustering of deaths by state. Finally, this study reports data from selected states and therefore does not represent the entire AI/AN population within the United States.

Despite these limitations, these findings suggest that, compared with the White population, the AI/AN population in the 14 participating states has been disproportionately affected by the COVID-19 pandemic, especially among younger age groups. Improved data quality and completeness for case investigation, death certificates, and laboratory reports can guide decisions on resource prioritization to identify and protect populations at higher risk for illness and death. Public health agencies should engage with tribes through tribal consultations and confer with urban-dwelling AI/AN communities to build upon existing community assets and values to enhance health outcomes. AI/AN communities have formed bidirectional partnerships with public health partners that are rooted in tribal sovereignty and fulfillment of treaty rights to promote culturally sensitive strategies for COVID-19 prevention activities and medical countermeasures (*10*).^†††^Strategies can draw on cultural factors that include protecting elders and ensuring a healthy future for younger generations. Improving the quality of COVID-19 data will be important for AI/AN communities and their partners to identify populations experiencing excess risk and plan and implement prevention activities and medical countermeasures.

**Summary**

**What is already known about this topic?**

COVID-19 incidence is higher among American Indians/Alaska Natives (AI/ANs) than among non-Hispanic Whites. In 2009, AI/ANs experienced disproportionately high pandemic influenza A(H1N1)–associated mortality.

**What is added by this report?**

Based on data from 14 participating states, age-adjusted COVID-19–associated mortality among AI/ANs was 1.8 times that among non-Hispanic Whites. Among AI/ANs, mortality was higher among men than among women, and the disparity in mortality compared with non-Hispanic Whites was highest among persons aged 20–49 years.

**What are the implications for public health practice?**

AI/ANs have experienced disproportionate rates of infection and mortality during the COVID-19 pandemic. The excess risk, especially for AI/AN males and persons aged 20–49 years, should be considered when planning and implementing medical countermeasures and other prevention activities.

## Footnotes

* Representatives from 14 state health departments, New York City Department of Health and Mental Hygiene, the Council of State and Territorial Epidemiologists, seven Tribal Epidemiology Centers, the Indian Health Service, and CDC.

† https://www.cdc.gov/nchs/nvss/vsrr/covid19/health_disparities.htm.

§ The following 14 states were solicited by CSTE to participate in the workgroup and provide data for analysis: Alaska, Arizona, Louisiana, Minnesota, Mississippi, Nebraska, New Mexico, New York, North Dakota, Oklahoma, Oregon, South Dakota, Utah, and Washington.

¶ https://data.census.gov/cedsci/table?q = 2019%20acs&tid = ACSDP1Y2019.DP05.

** AI/AN was defined as AI/AN alone or multiracial and includes non-Hispanic and Hispanic ethnicity. White was defined as non-Hispanic ethnicity with a response of White only and no other race selected.

†† Data were analyzed and confirmed separately by the Urban Indian Health Institute and the Northwest Tribal Epidemiology Center.

§§ https://www.census.gov/content/census/en/data/datasets/time-series/demo/popest/2010s-state-detail.html.

¶¶ 45 C.F.R. part 46.102(l)(2).

*** https://cdn1.sph.harvard.edu/wp-content/uploads/sites/1266/2020/06/20_Bassett-Chen-Krieger_COVID-19_plus_age_working-paper_0612_Vol-19_No-3_with-cover.pdf.

††† Some authors have been engaged to support the development of data use agreements with tribes, their state public health department, and their Tribal Epidemiology Center to promote timely, culturally responsive case investigation, contact tracing, and wraparound services for members of their communities affected by COVID-19.

## References

[1] CDC. Deaths related to 2009 pandemic influenza A (H1N1) among American Indian/Alaska Natives—12 states, 2009. MMWR Morb Mortal Wkly Rep 2009;58:1341–4. 20010508

[2] Hennessy TW, Bruden D, Castrodale L, ; Investigative Team. A case-control study of risk factors for death from 2009 pandemic influenza A(H1N1): is American Indian racial status an independent risk factor? Epidemiol Infect 2016;144:315–24. 10.1017/S0950268815001211 26118767 PMC5222627

[3] Hatcher SM, Agnew-Brune C, Anderson M, COVID-19 among American Indian and Alaska Native persons—23 states, January 31–July 3, 2020. MMWR Morb Mortal Wkly Rep 2020;69:1166–9. 10.15585/mmwr.mm6934e1 32853193 PMC7451969

[4] Kakol M, Upson D, Sood A. Susceptibility of Southwestern American Indian tribes to coronavirus disease 2019 (COVID-19). J Rural Health 2020. Epub June 1, 2020. 10.1111/jrh.12451 32304251 PMC7264672

[5] Mamelund SE, Shelley-Egan C, Rogeberg O. The association between socioeconomic status and pandemic influenza: protocol for a systematic review and meta-analysis. Syst Rev 2019;8:5. 10.1186/s13643-018-0931-2 30609940 PMC6318944

[6] Fiore AE, Uyeki TM, Broder K, . Prevention and control of influenza with vaccines: recommendations of the Advisory Committee on Immunization Practices (ACIP), 2010. MMWR Recomm Rep 2010;59(No.RR-8). 20689501

[7] McClung N, Chamberland M, Kinlaw K, The Advisory Committee on Immunization Practices’ ethical principles for allocating initial supplies of COVID-19 vaccine—United States, 2020. MMWR Morb Mortal Wkly Rep 2020;69:1782–6. 10.15585/mmwr.mm6947e3 33237895 PMC7727606

[8] Weinberger DM, Chen J, Cohen T, Estimation of excess deaths associated with the COVID-19 pandemic in the United States, March to May 2020. JAMA Intern Med 2020;180:1336–44. 10.1001/jamainternmed.2020.3391 32609310 PMC7330834

[9] Arias E, Heron M, Hakes J. The validity of race and Hispanic-origin reporting on death certificates in the United States: an update. Vital Health Stat 2 2016;172:1–21. 28436642

[10] Kuhn NS, Sarkar S, White LA, Decolonizing risk communication: indigenous responses to COVID-19 using social media. J Indig Soc Dev 2020;9:193–213.

