# *Erratum:* Vol. 69, No. 49

**DOI:** 10.15585/mmwr.mm7001a6

**Published:** 2021-01-08

In the report “COVID-19 Mortality Among American Indian and Alaska Native Persons — 14 States, January–June 2020,” on page 1853, the list of authors should have read “Jessica Arrazola, DrPH^1^; Matthew M. Masiello^1^; Sujata Joshi, MSPH^2^; Adrian E. Dominguez, MS^3^; Amy Poel, MPH^3^; Crisandra M. Wilkie, MPH^3^; Jonathan M. Bressler, MPH^4^; Joseph McLaughlin, MD^4^; Jennifer Kraszewski, MS^5^; Kenneth K. Komatsu, MPH^6^; Xandy Peterson Pompa, MPH^6^; Megan Jespersen, MPH^7^; Gillian Richardson, MPH^7^; Nicholas Lehnertz, MD^8^; Pamela LeMaster, PhD^8^; Britney Rust, MPH^9^; Alison Keyser Metobo, MPH^10^; Brooke Doman, MPH^11^; David Casey, MA^12^; Jessica Kumar, DO^12^; Alyssa L. Rowell, MA^12^; Tracy K. Miller, PhD^13^; Mike Mannell, MPH^14^; Ozair Naqvi, MS^14^; Aaron M. Wendelboe, PhD^14^; Richard Leman, MD^15^; Joshua L. Clayton, PhD^16^; Bree Barbeau, MPH^17^; Samantha K. Rice, MPH^18^; **Samantha J.H. Rolland, PhD^18^**; Victoria Warren-Mears, PhD^2^; Abigail Echo-Hawk, MA^3^; Andria Apostolou, PhD^19^; Michael Landen, MD^1^.

The affiliation for Samantha J.H. Rolland is Washington State Department of Health.

